# Detection and Classification of Knee Osteoarthritis

**DOI:** 10.3390/diagnostics12102362

**Published:** 2022-09-29

**Authors:** Joseph Humberto Cueva, Darwin Castillo, Héctor Espinós-Morató, David Durán, Patricia Díaz, Vasudevan Lakshminarayanan

**Affiliations:** 1 Departamento de Química, Facultad de Ciencias Exactas y Naturales, Universidad Técnica Particular de Loja, San Cayetano Alto s/n, Loja 11-01-608, Ecuador; 2 Instituto de Instrumentación para Imagen Molecular (i3M) Universitat Politècnica de València—Consejo Superior de Investigaciones Científicas (CSIC), 46022 Valencia, Spain; 3 Theoretical and Experimental Epistemology Lab, School of Optometry and Vision Science, University of Waterloo, Waterloo, ON N2L3G1, Canada; 4 Escuela de Ciencia, Ingeniería y Diseño, Universidad Europea de Valencia, Paseo de la Alameda 7, 46010 Valencia, Spain; 5 Applied Data Science Lab (ADaS Lab), Facultat Informàtica, Multimedia i Telecomunicacions, Universitat Oberta de Catalunya, Avenida Tibidabo 39-43, 08035 Barcelona, Spain; 6 Facultad de Ciencias Médicas, Universidad Técnica Particular de Loja, San Cayetano Alto s/n, Loja 11-01-608, Ecuador; 7 Departments of Physics, Electrical and Computer Engineering and Systems Design Engineering, University of Waterloo, Waterloo, ON N2L3G1, Canada

**Keywords:** CAD, OA knee, osteoarthritis, CNN, X-ray images, KL grades, deep learning

## Abstract

Osteoarthritis (OA) affects nearly 240 million people worldwide. Knee OA is the most common type of arthritis, especially in older adults. Physicians measure the severity of knee OA according to the Kellgren and Lawrence (KL) scale through visual inspection of X-ray or MR images. We propose a semi-automatic CADx model based on Deep Siamese convolutional neural networks and a fine-tuned ResNet-34 to simultaneously detect OA lesions in the two knees according to the KL scale. The training was done using a public dataset, whereas the validations were performed with a private dataset. Some problems of the imbalanced dataset were solved using transfer learning. The model results average of the multi-class accuracy is 61%, presenting better performance results for classifying classes KL-0, KL-3, and KL-4 than KL-1 and KL-2. The classification results were compared and validated using the classification of experienced radiologists.

## 1. Introduction

Research in osteoarthritis (OA) pathology has been necessary for years due to its high economic impact, disability, pain, and severe impact on the patient’s lifestyle. OA goes beyond anatomical and physiological alterations (joint degeneration with gradual loss of joint cartilage, bone hypertrophy, changes in the synovial membrane, and loss of joint function) since cellular stress and degradation of the extracellular cartilage matrix begin with micro-and macro-injuries [1]. Generally, OA is associated with aging. However, there are other risk factors namely obesity, lack of exercise, genetic predisposition, bone density, occupational injury, trauma, and gender [2,3].

OA affects nearly 240 million people worldwide [4]. According to the World Health Organization (WHO), by 2050, approximately 20% of the world’s population will be over 60 years old. Of that percentage, 15% will have symptomatic OA, and one-third of these people will be severely disabled [1,2,3,4].

Knee osteoarthritis (KOA) or knee joint disease is the most common type of arthritis diagnosed, especially in older adults [2]. The population suffering from OA presents symptoms such as chronic pain, crepitus, edema, morning stiffness, atrophy, decreased quadriceps muscle strength, and impaired postural control, which causes difficulty in performing the usual activities of daily living [5].

Osteoarthritis is currently diagnosed by physical examination and commonly also through images of X-ray, MRI scan, and arthroscopy [2]. However, these diagnostic tools have low sensitivity and specificity due to the high level of subjectivity [6] and dependency on the experience of the clinician making the diagnosis. The MRI technique has several limitations, such as the expensive cost, and it depends on the chondral anatomical location and the evaluating physician. Due to these factors, on many occasions the severity of the chondral lesion is underestimated; observing that only 30% of the MRIs showed an adequate cartilaginous state in all the anatomical locations.

Physicians measure the severity of knee OA according to the Kellgren and Lawrence (KL) [7] grading system developed for visual inspection of X-ray images or MRI [4,7]. The KL system splits knee OA severity into five grades from grade 0 (normal) to grade 4 (severe) [6]. Hence, in MRI, the sensitivity oscillates between 92% in healthy cartilage and 5% in grade I lesions. Specificity also varies according to the grade of the lesion, reaching 96.5% in grade IV lesions and 38% in healthy cartilage [8].

Epidemiological studies manifest that due to the exponential growth in the prevalence of OA, the health system is saturated, requiring a slow and repetitive process for the diagnosis and to follow the evolution of the disease [6]. However, over the years and based on the magnitude of the disease, other evaluation techniques have been developed, such as shear wave elastography (SWE) [9].

SWE is a non-invasive technique, free of radiation, whose objective is to evaluate the elasticity of the soft tissues. In OA, the atrophied function of the quadriceps femoris muscle aggravates the pain of the knee and gradually worsens the mechanical function of the quadriceps femoris [9].

Another technique for early detection of OA is vibroarthrography (VAG). This reproducible, inexpensive, radiation-free, easy, and accessible tool takes advantage of the vibrations and sounds generated by the joint during flexion-extension motion [10]. A healthy, lubricated joint moves silently, while a pathologic joint with poorly lubricated, rough articular surfaces generates incongruent motion and a readily noticeable sound (crepitus) [11]. These vibratory signals in motion are generated by transient elastic waves resulting from redistribution of joint material and can be recorded from the knee surface. The published sensitivity and specificity range from 0.56–1 and 0.74–1, respectively [8].

In search of the simplest and most effective diagnosis without invasive methods, science has combined parameters such as VAG, the support vector machine (SVM), and the Bayesian decision rule to perform the VAG signal classifications. The classification experiment results demonstrate that the Bayesian decision rule can produce an overall classification accuracy of 84%, with a sensitivity value of 0.75 and a specificity value of 0.894 [12].

Due to the high prevalence of OA, it is necessary to develop new methods and technologies to measure OA progression, grading, and detection [4]. In this context, the development of algorithms and applications of Machine Learning (ML) and Deep Learning (DL) would help physicians to get diagnoses and biomarkers to measure the status and progression of OA more effectively by performing medical image analysis through automatic or semi-automatic systems [2].

It is convenient to optimize the diagnosis through a computer-assisted approach based on machine learning capable of solving the diagnostic problem through automatic X-ray diagnosis; some of these models reach a detection of 98.516% and a classification accuracy of 98.90% [13].

In the case of knee OA, e.g., Shamir et al. [7] proposed a technique with a sliding window strategy to locate the knee joints. Antony et al. [14] proposed a fully convolutional network (FCN) system to detect knee joints and for the automated quantification of the severity of KOA according to the KL scale, which obtained a better classification accuracy with KL grades 3 and 4 than grades 0, 1 and 2 due to the slight variations in the image(s).

Thomson et al. [15] employed shape and texture analysis combined with a random forest classifier in the radiograph knee, with an AUC of 0.849. Chen et al. [4] proposed a method that combined two deep convolutional neural networks to detect and classify knee joints according to the KL scale with a classification accuracy of 69.7% using the fine-tuned VGG-19 [16] model compared with ResNet [17] or DenseNet [18]. The approach of Tiulpin et al. [3] to learn the symmetry of a pair of knee X-ray images and identify similarity metrics between the lateral section and the medial section of a knee is based on the Deep Siamese network architecture that was implemented by Chopra et al. [19]. Their results show an average multiclass KL accuracy of 66.71% through a Deep Siamese convolutional neural network model.

Another combination with good results is using artificial neural networks (ANN) of RBF (Radial Basis Function) and MLP (Multilevel Perceptron) types. An accuracy of 0.9 was obtained, with a sensitivity of 0.885 and a specificity of 0.917. It has been shown that vibroacoustic diagnostics have great potential in the non-invasive assessment of damage to joint structures of the knee [20].

One common problem with the use of deep learning is the availability of sufficient data for the training and validation of the models [21]. In the case of OA, there are only a few public standard datasets, such as the Multicenter Osteoarthritis Study (MOST) [22] and the Osteoarthritis Initiative (OAI) [23].

Here we present a computer-assisted diagnostic (CAD) system to detect and automatically classify knee OA by processing X-ray images and providing the KL grade. The proposed model is based on the Deep Siamese convolutional neural networks and a fine-tuned ResNet-34 to detect lesions in the two knees simultaneously. The problem of the imbalanced dataset is solved using transfer learning. Training of the model is done with a public dataset, whereas a private dataset was used for validation. The model results of the classification are compared with the literature and the diagnosis of experienced radiologists. The multiclass accuracy of the model is 61%.

## 2. Materials and Methods

### 2.1. Dataset

Different collections of knee X-ray images labeled according to the Kellgren–Lawrence (KL) scale were utilized in this work (see Table 1). The dataset used for training and validating the proposed model was obtained from Chen et al. [4]. The dataset contains knee X-ray data with knee KL grading; the dataset is organized from OA [14]. The dataset is a multi-center, longitudinal, prospective observational study of knee osteoarthritis (KOA) aiming to identify biomarkers for OA onset and progression. This dataset [4] consists of 4796 participants ranging in age from 45 to 79 years. The dataset contains about nine thousand images, with the KL-0 class having the largest number of images and KL-4 the smallest number of images.

For testing the model, a dataset composed of 376 knee X-ray images from a private hospital was used [24]. The dataset was classified according to the KL scale by clinical experts in OA pathology.

### 2.2. Preprocessing

The number of images from Chen et al. [4] dataset varies according to the KL scale; hence, it presents a class imbalance problem. The effect of class imbalance on classification is detrimental to the performance of the model [25]. To improve the class imbalance, we applied oversampling to the minority classes of the dataset based on the concepts exposed by Buda et al. [25]. In addition, new training data were created from existing data through data augmentation techniques [26].

Image data augmentation involves transformed versions of the original image belonging to the same class. The transformation applied to the images was a random rotation between −7 to 7 degrees, and the color jitter randomly changed the images’ brightness, contrast, and saturation [26]. All images that could not be seen after the transformation was discarded.

After preprocessing, 90% of this image set was used for model training and the rest for validation. In the test dataset, images with a knee prosthesis or treatment of bone fracture at the knee level were discarded. For a balanced dataset, 45 images of each class were taken, giving a total of 225 images (see Table 1).

### 2.3. Network Architecture

**Figure 1:**
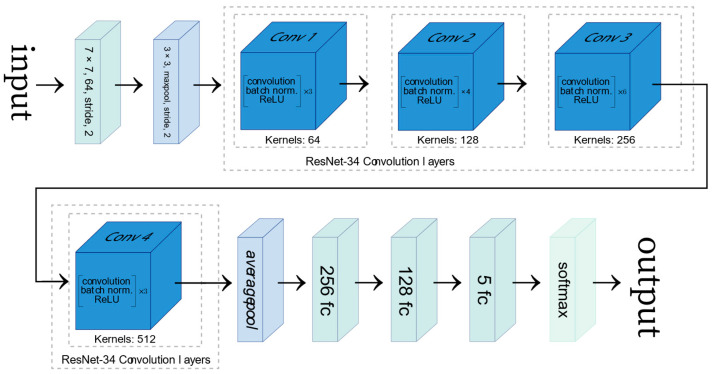
Representation of the ResNet-34 architecture modified with two fully connected (FC) layers added. The figure was adapted from [28].

The convolutional neural network used in this work is based on a fine-tuned ResNet-34 [17], which was pre-trained on the ImageNet database [27]. Since the ResNet-34 was trained to classify images amongst 1000 different classes, the original model’s last fully connected (FC) layer was modified, and two extra FC layers were added in order to employ the transfer learning methodology (see Figure 1). Since the ResNet-34 network was trained with color images (RGB) for computer vision tasks, the training process was adapted as X-ray images are greyscaled. Therefore, the grayscale channel of the X-ray image was taken as input for each of the channels of an RGB image.

**Figure 2:**
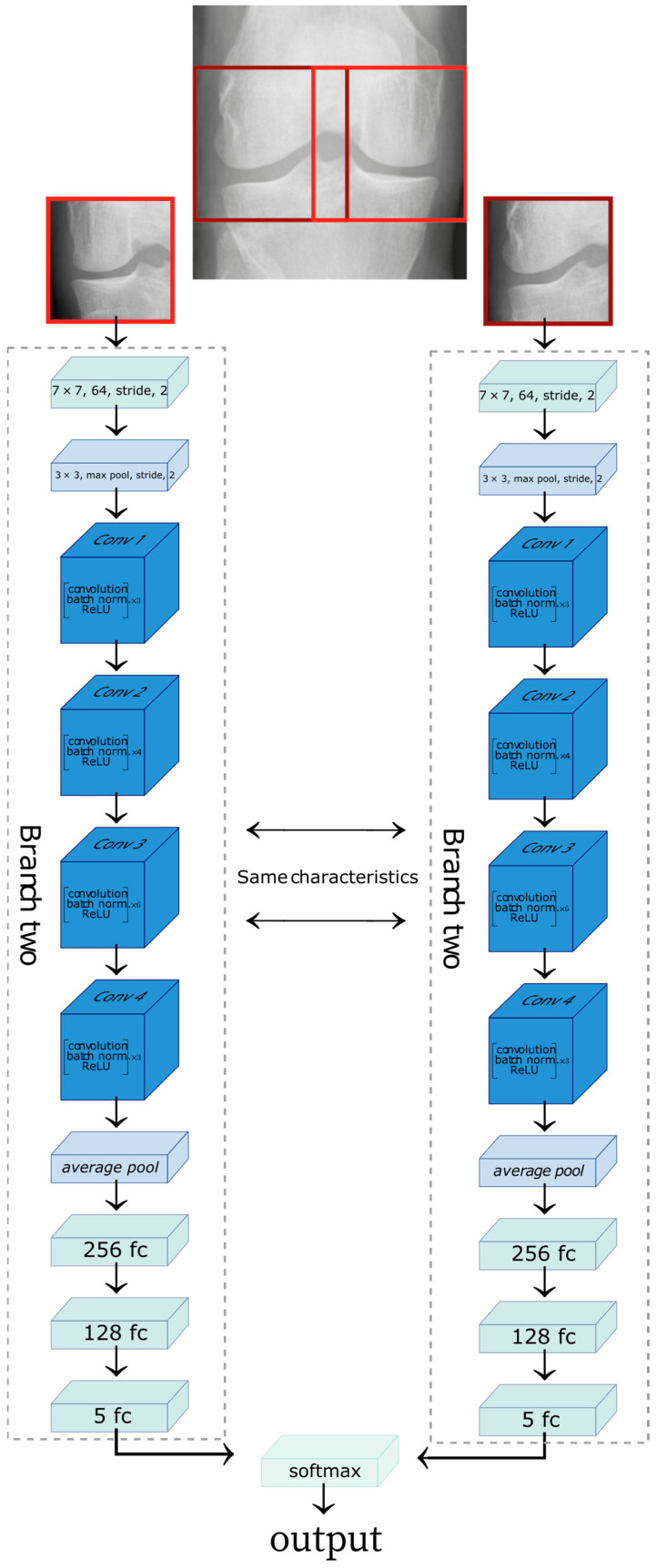
Representation of the Siamese network architecture. The lateral side of the knee X-ray image feeds one branch of the network, and the medial side of the knee X-ray image provides the second branch of the network. The figure was adapted from [28].

To identify similarity metrics between the lateral and medial sections of a knee, we used the Deep Siamese network [2] to allow learning the characteristics of knee osteoarthritis and the level of disease. The images were cropped on the lateral and medial sides. The medial side image was horizontally flipped to maintain symmetry with the lateral image. The Deep Siamese CNN was fed a pair of images: the image of the lateral section provides one branch of the network while the image of the medial compartment was fed to the other, as shown in Figure 2.

The two branches of the network are composed of the same stages. Each branch would learn the exact characteristics of the input images. Each branch consists of a convolution network; the first step on the convolution network before entering ResNet layers (blue boxes) consists of a convolution, batch normalization, and max-pooling operation. Each ResNet layer comprises multiple blocks, consisting of convolution, batch normalization, and a ReLU to an input.

**Figure 3:**
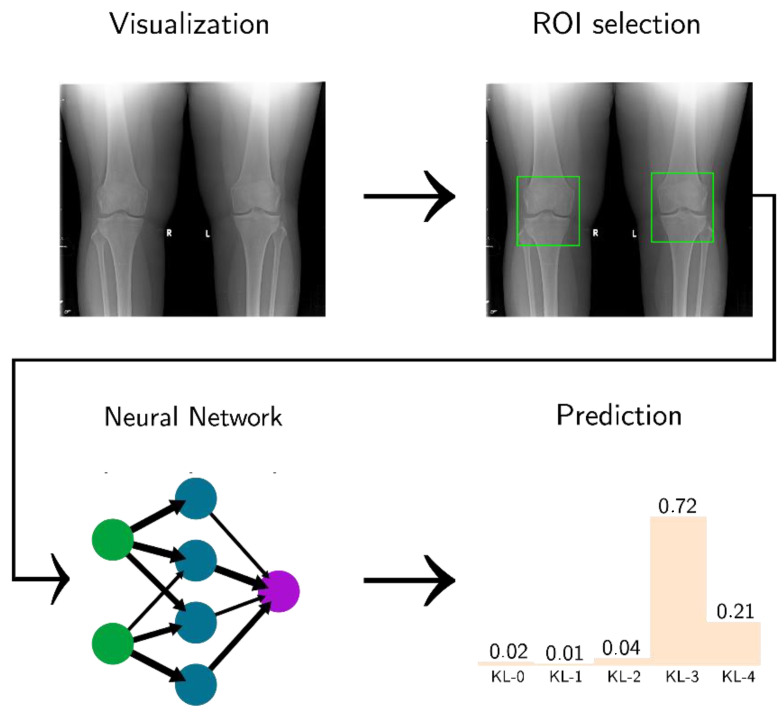
Software pipeline used in this project. The figure was adapted from [29].

The ResNet layer is followed by an average pool block, which is the input of the FC layers. At the end of each branch, we have a map feature of input, and the result from the two networks are concatenated. The final block is a SoftMax, which returns the model result as a distribution of probabilities between 0 and 1 for each KL class (see Figure 3).

## 3. Results

### 3.1. Classification of KL Degrees

The metrics used to analyze the results are confusion matrix, accuracy, precision, and recall.

**Figure 4:**
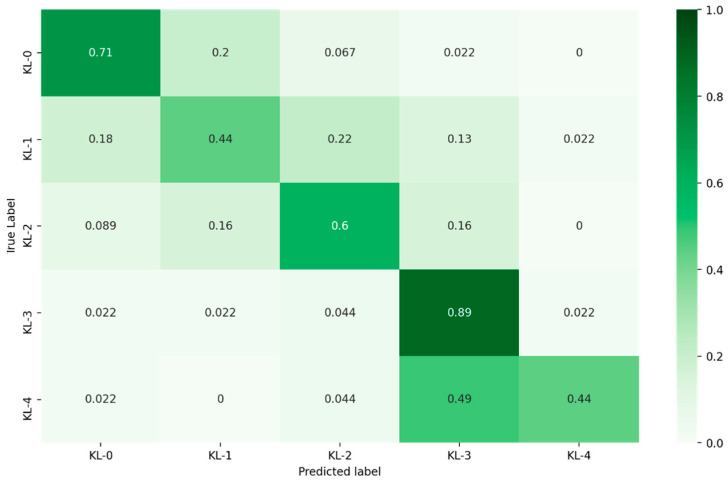
Confusion matrix for KL grading according to the proposed model.

The proposed model was tested and evaluated using the private clinic dataset. The confusion matrix for the KL grading results is presented in Figure 4. Table 2 summarizes the number of the true positive (TP), false positive (FP), and false negative (FN) values, precision, recall for each KL class and the execution time of the model to classify all the test dataset images.

**Figure 5:**
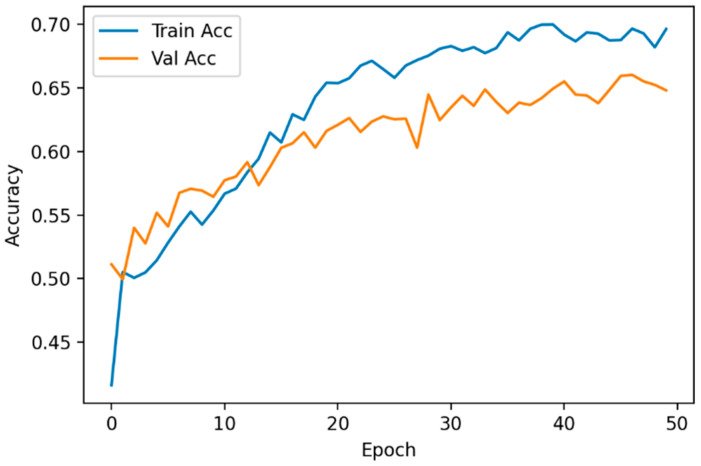
Evolution of the accuracy for the training set and the validation set.

For the implementation and training of the ResNet-34, the Pytorch framework and an Nvidia Tesla P-100 GPU card were utilized. The model was trained for 50 epochs, detecting overfitting at 40 epochs. To reduce the overfitting, the training data were increased through data augmentation. As a result, the overfitting no longer occurred after 40 epochs but instead started at epoch 50. To reduce overfitting without compromising the model accuracy (Figure 5), early stopping was applied when the validation accuracy did not improve after 50 epochs

### 3.2. KL Model Comparison

**Figure 6:**
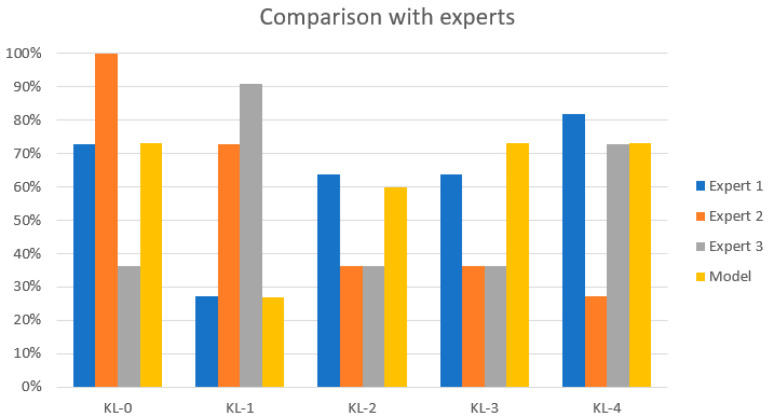
Comparison with the experts. The yellow bar represents the classification results of the proposed model. There is a low classification accuracy for KL-1 and KL-2 grades.

The performance of the proposed model was compared with the diagnosis of experienced radiologists and clinicians from different hospitals in Ecuador. Each clinician labeled 11 knee X-ray images from the test dataset. The results are shown in Table 3 and Figure 6. The results obtained by the model are close to the assessment of the experts with the highest rate of diagnosis agreement in classes KL-0, KL-3, and KL-4. The performance for the KL-1 class is closer to the assessment of the expert with the lowest diagnosis rate. The results for the KL-2 type are between the highest rate of diagnosis and the lowest rate of diagnosis.

Table 4 compares the results obtained in this work with other published studies. The table collects different metrics reported by the authors, including average multiclass accuracy, kappa coefficient, and network training information. As shown, the results obtained by our model outperform the results of Antony et al. [14]. Mainly, our model performs with a higher accuracy and kappa coefficient for classification but does not achieve the results obtained by Tiulpin et al. [3] and Zhang et al. [29].

### 3.3. Classification and User Interface

**Figure 7:**
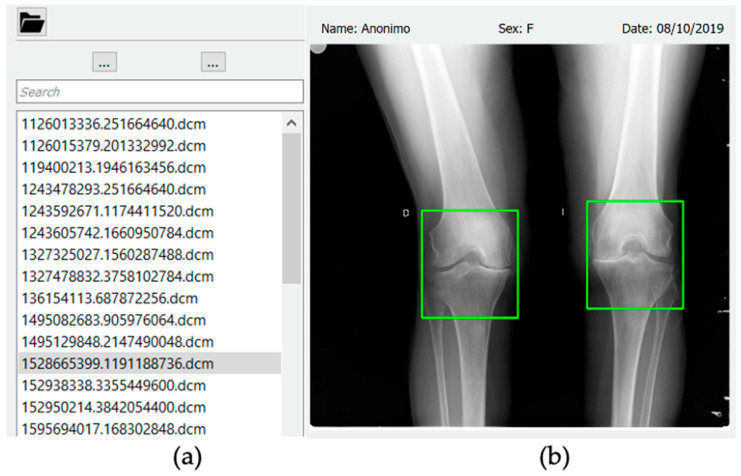
(**a**) Navigation panel, that allows selection of the image to process (*.dcm files). (**b**) The X-ray image panel where the region of interest is selected.

A graphic interface has been developed where X-ray images can be loaded and displayed. To process an X-ray image of the knee, users must first select directory in the upper navigation bar. Once the directory is selected, all the *.dcm files will be loaded in the navigation panel (Figure 7a).

When a file from the navigation panel is selected, it will be displayed in the X-ray image panel. By clicking the ROI button, two boxes will be drawn on the displayed X-ray image panel. Users can move the boxes to select the joint they wish to analyze. Once the region of interest is selected, users must click on the process button, and in a few seconds the results of the model will be displayed as shown in Figure 7b.

**Figure 8:**
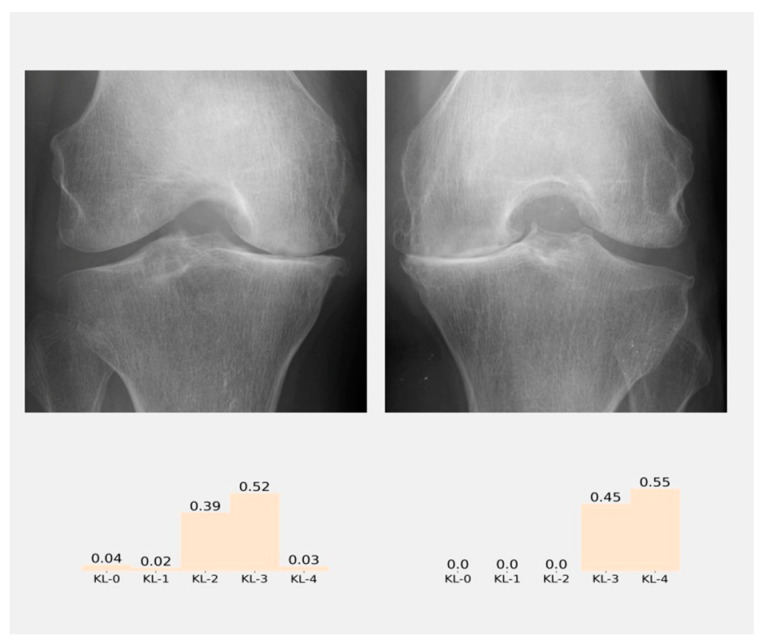
Result panel where the probabilities of the classification according to KL grades in each knee of the patient are shown.

**Figure 9:**
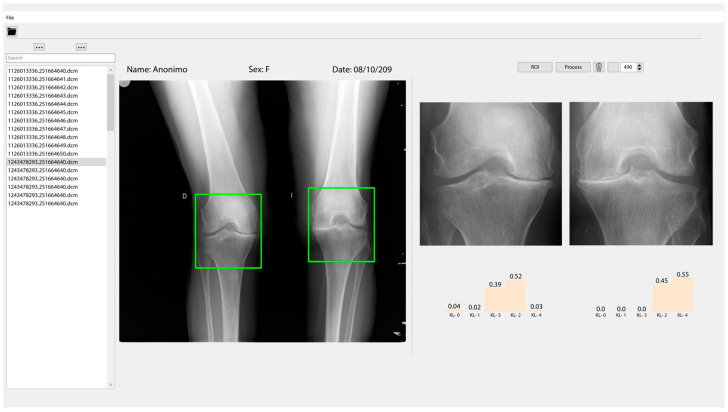
The graphic interface where the classification of severity of the knee osteoarthritis (KOA) is shown according to the KL grades from the radiographic image.

The results of classification will appear in the results panel as a distribution of probabilities (Figure 8), and the class with the higher probability is the class predicted by the model. Figure 9 shows all the graphic interface developed

## 4. Discussion and Conclusions

In this project, an approach for automatically diagnosing and grading knee OA from plain radiographic images is presented and integrated with a graphical interface for manually selecting the region of interest.

Table 4 details the values of multiclassification compared with similar works [3,15,29]. Our method achieves an average multi-class accuracy of 61.71%, which is a good value, especially with the classification of classes KL3 and KL 4.

The model seems to perform poorly in classifying KL-1 and KL-2 classes. This effect is a limitation in the model developed. However, it is susceptible to improvement in future works with better image preprocessing techniques and a better curation of the dataset. This is due principally to the fact that the X-ray images are operant dependent and also there is a slight difference between classes KL-1 and KL-2.

The interface developed gives the possibility to the physician to select the best region of interest according to the best professional criteria. In addition, as stated by Zhang et al. [29], there are intrinsic difficulties in distinguishing class KL-1 from KL-0 and KL-2 even for experienced specialists. This last point can be seen in Table 3, e.g., the average classification from experts in KL-1 and KL-2.

However, the model presents similar results for classifying classes KL-0, KL-3, and KL-4 compared to radiologists (see Table 3). Nevertheless, the model obtains a good accuracy, and kappa scores outperform previous studies reported (see Table 4); the model can learn relevant features of OA and that learning can be transferred to different data sets.

The fact that compressed images were utilized for training may constitute a limitation as it could have led to the loss of information present in the images; hence, a higher resolution of the original images could improve our results, but of course this also increases the computational cost. Moreover, potentially misclassified images in the training dataset could somewhat affect the model’s performance. In addition, our model could be enhanced by using more significant amounts of training images with a broader group of experts for the classification.

In this context, X-ray images have some advantages such as economic costs and accessible technology, even in low-income countries. However, it is fundamental to take care of the alignment of the X-ray beam and the orientation of the patellofemoral compartment for the diagnosis by radiographic images; these details help to identify the narrowing of the articular space (thickness of the articular cartilage), osteophytes and sclerosis of the subchondral bone [10].

Currently, the KL classification is the most used clinical tool for the radiographic diagnosis of OA [30]. Another essential factor to consider for medical decision-making through X-ray images is the sensitivity and specificity of this KL scale which reaches 90% and 24.6% [31], respectively.

The significant variability of symptoms in OA delays and hinders diagnosis. However, pain is the main symptom requiring medical attention; pain also has a direct association with radiological changes, as a study of 4680 participants [32] reported that 78.8% of participants with OA felt pain, as diagnosed by X-ray.

The latter represents a moderate value in the early stages of joint injury, so the clinical aspect is needed to reach the diagnosis [33]. This radiographic-clinical disagreement may be manifested by the multifactorial origin of pain in each patient and the pain tolerance of each individual [32]. It is here that data processing plays a crucial role in optimizing time and reaching consensus on the different medical criteria.

Finally, the developed CAD constitutes a tool for training young specialists and medical students. In future works, we plan to present results about the processing of medical images with clinical data, which means working on a better personalization of the treatment and diagnosis.

## Figures and Tables

**Table 1:** Description of the raw dataset and the processed dataset. The numbers in the tables indicate the number of knee images used in each group.

Group	Dataset	Images	KL-0 ^1^	KL-1	KL-2	KL-3	KL-4
Raw dataset	Chen et al., 2019 [4]	9182	3253	1770	2578	1286	285
Raw dataset	Private hospital	376	58	65	95	113	45
Training	Chen et al., 2019 [4]	20,022	4422	4395	4262	4648	2295
Validation	Chen et al., 2019 [4]	1359	270	270	270	270	270
Test	Private hospital	225	45	45	45	45	45

^1^ Kellgren–Lawrence grading system, KL-0 (normal) to grade KL-4 (severe).

**Table 2:** Summary of TP, FP, and FN values for each KL class.

Kellgren–Lawrence Scale	TP	FP	FN	Precision	Recall	Execution Time
KL-0	32	14	13	70%	71%	6.11 s
KL-1	20	17	25	54%	44%	
KL-2	27	17	18	61%	60%	
KL-3	40	36	5	53%	89%	
KL-4	20	2	25	91%	44%	

**Table 3:** Model performance comparison with expert assessment.

Kellgren–Lawrence Scale	Expert 1	Expert 2	Expert 3	Our Model
KL-0	73%	100%	36%	73%
KL-1	27%	73%	91%	27%
KL-2	64%	36%	36%	50%
KL-3	64%	36%	36%	73%
KL-4	82%	27%	73%	73%

**Table 4:** Model comparison to other models according (kappa an average multiclass accuracy).

Model	Learning Rate	Optimizer	Kappa	Average Multiclass Accuracy
This work	1 × 10^−4^	Adam	0.79	61.71%
Tiulpin et al., 2018 [3]	1 × 10^−4^	Adam	0.83	66.71%
Antony et al., 2017 [14]	1 × 10^−3^	SGD	0.77	59.52%
Zhang et al., 2020 [29]	-	-	0.88	74.81%

## Data Availability

Software developed and private data used in this project is available at: https://drive.google.com/drive/folders/1NljuU_nZB0R4UVVizk3kXwv10Tsr_ukr?usp=sharing (accessed on 12 June 2022); https://github.com/jhcueva/OA-Analyzer (accessed on 12 June 2022).

## Abbreviations

AI	Artificial intelligence
OA	Osteoarthritis
KL	Kellgren and Lawrence scale
AUC	Area under the curve
CNN	Convolutional neural network
CAD	Computer-aided diagnosis/detection
DL	Deep learning
